# The effect of intravesical instillation of antifibrinolytic agents on bacillus Calmette-Guerin treatment of superficial bladder cancer: A pilot study

**Published:** 2008

**Authors:** Pawan Vasudeva, Dharamveer Singh, Apul Goel

**Affiliations:** Department of Urology, C.S.M.M.U (Upgraded King George Medical College), Lucknow, Uttar Pradesh, India. E-mail: pv_22@yahoo.com

## SUMMARY

The authors in this study have assessed the impact of concomitant intravesical administration of antifibrinolytic agents on the antitumor effect of bacillus Calmette-Guerin (BCG) in postoperative patients with superficial bladder cancer (SBC). The impact of antifibrinolytic agents [para-aminomethylbenzoic acid (PAMBA) and epsilon aminocaproic acid (EACA)] on the effective dose of BCG was also investigated.

From October 1999 to December 2004, 257 patients with transitional cell carcinoma of the bladder who had undergone either transurethral resection of bladder tumor (TURBT) or partial cystectomy and had SBC as confirmed by biopsy were included in this randomized, prospective, double-blind, controlled study. Exclusion criteria included previous history of BCG instillation or vaccination, hematological disease or tuberculosis. Among these patients, 180 were men, median age was 58.5 years, 188 had primary tumors and 209 had a single tumor. The majority had low to intermediate grade tumors. All received intravesical therapy using a similar protocol of six weekly instillations followed by monthly instillations for two years. Patients were randomized into five groups from A to E. They received 100 to 120 mg BCG plus 100 mg PAMBA, 50 to 60 mg BCG plus 100 mg PAMBA, 100 to 120 mg BCG plus 2.0 gm EACA, 50 to 60 mg BCG plus 2.0 gm EACA and 100 to 120 mg BCG alone, respectively. Prothrombin time (PT) and activated partial thromboplastin time (APTT) measurements were done 2 h after BCG instillation and adverse events were evaluated. Tumor recurrence was assessed every three months postoperatively. Median follow-up was 25 months. Twenty-five patients withdrew due to serious side-effects and 13 patients were lost to follow-up. Patient and tumor characteristics were similar among all the groups. No significant change in PT or APTT was observed in any patient group. A total of 32 patients experienced recurrence. Recurrence rates at a minimum of median two years were 10.6%, 11.1%, 10.0%, 9.3% and 31.8% in Groups A through E, respectively. Recurrence rates were statistically insignificant when pair-wise comparison was done among Groups A, B, C and D. In contrast, patients in Group E had a statistically significant higher rate of recurrence when pair-wise comparison was done with Groups A,B,C and D (*P* =.019,.036,.018,.016 respectively). Recurrence-free probability also showed a similar trend and it was significantly lower for patients in Group E when pair-wise comparison was done with the other groups. The rate of serious adverse events in Groups A through E was 9.6%, 3.9%, 15.7%, 5.9% and 13.5%, respectively and these were statistically insignificant. Based on the above findings, the authors concluded that concomitant intravesical instillation of antifibrinolytic agents such as PAMBA and EACA with BCG can safely improve the antitumor effect of BCG even with a reduced BCG dose.

## COMMENTS

Following TURBT, 30-85% of patients with SBC will develop recurrent disease and nearly 20-30% are at risk for disease progression. BCG immunotherapy decreases tumor recurrence, disease progression and bladder cancer-specific mortality. This article has tried to address two issues regarding BCG immunotherapy. First is the controversy that exists about the optimum dosage of BCG. Though lower dosage is associated with lesser side-effects, there are still doubts about its efficacy as compared to standard doses, especially in high-risk cases. Second is regarding the need for improving the efficacy of BCG therapy as such in light of the considerable number of patients who do develop BCG failures.

The rationale behind concomitant administration of antifibrinolytics with intravesical BCG is evident from the understanding of the antitumor mechanisms of intravesical BCG therapy. Though the exact mechanisms are still being elucidated, the first prerequisite step is attachment of the BCG to the bladder wall. This attachment is essential for the antitumor response and is mediated via the extracellular matrix fibronectin. [1] Fibronectin, a ubiquitous and abundant extracellular, insoluble matrix protein, is secreted by cells in a soluble dimeric form and cell-mediated processes convert it into the extracellular insoluble form. Increased blood coagulation enhances this conversion. [2,3] Since the extent of attachment correlates with the antitumor activity, addition of antifibrinolytic agents may potentially enhance tumor response by increasing matrix fibronectin and thereby BCG attachment. Animal experiments have shown that intravenous/intravesical administration of antifibrinolytic agents enhances BCG attachment and antitumor efficacy. [4,5] This is the first prospective clinical study of its kind in humans. Though the results are promising, whether this treatment would become a standard means of safely improving the antitumor effect of BCG is something that would need more clinical trials for clarification.
